# Fokker-Planck analysis of optical near-field traps

**DOI:** 10.1038/s41598-019-45609-x

**Published:** 2019-07-02

**Authors:** Mohammad Asif Zaman, Punnag Padhy, Lambertus Hesselink

**Affiliations:** Stanford University, Electrical Engineering, Stanford, CA 94305 USA

**Keywords:** Optical manipulation and tweezers, Nanophotonics and plasmonics

## Abstract

The motion of a nanoparticle in the vicinity of a near-field optical trap is modeled using the Fokker-Planck equation. A plasmonic C-shaped engraving on a gold film is considered as the optical trap. The time evolution of the position probability density of the nanoparticle is calculated to analyze the trapping dynamics. A spatially varying diffusion tensor is used in the formulation to take into account the hydrodynamic interactions. The steady-state position distribution obtained from the Fokker-Planck equation is compared with experimental results and found to be in good agreement. Computational cost of the proposed method is compared with the conventionally used Langevin equation based approach. The proposed method is found to be computationally efficient (requiring 35 times less computation time) and scalable to more complex lab-on-a-chip systems.

## Introduction

Techniques of manipulating micron and submicron sized particles using non-contact forces are an important area of research. Optical trapping techniques, pioneered by Arthur Ashkin in the early 1970s, employ optical forces to trap and manipulate particles^1–3^. The single beam optical gradient force trap, or *optical tweezers*, is one of the most widely used schemes in the field of biology^4,5^. In addition to bio-technology, optical traps have found applications in the fields of physics^6,7^ and chemistry^8^. Near-field optical trapping methods, such as *plasmonic tweezers*^9^ have also been developed to trap submicron sized particles^10–15^. With the development of complex lab-on a chip (LOC) systems where multiple near-field traps are fabricated on the same chip, accurately modeling the motion of nanoparticles in a trapping force field is becoming more and more important.

In a conventional optical tweezer, a laser beam is focused through an objective lens to exert gradient forces on a particle to trap it. The smallest particle that can be trapped using this technique is determined by the minimum possible optical spot size and hence the diffraction limit. An alternative method is required to trap nanoparticles smaller than what the diffraction limit permits. Near-field trapping schemes use plasmonic structures^16,17^ or dielectric waveguides^11^ to create strong localized intensity enhancements. This can create a focused spot size smaller than the diffraction limit, and thus allowing the trapping of smaller particles. In addition to having the capacity to trap nanoparticles, near-field traps have a geometry that is suitable for parallelization. The planar geometry of plasmonic structures makes it possible to pack multiple near-field traps on the same chip and to perform controlled complex manipulations^18,19^. A common light source with a relatively large spot size can be used to excite multiple of such traps simultaneously. For these reasons, plasmonic near-field traps are suitable candidates for use in LOC systems^20–24^.

To design LOC systems that contain near-field traps, it is necessary to model and analyze the trapping dynamics. The motion of a nanoparticle under the influence of a trapping force-field must be understood in order to design a platform that aims to achieve any kind of complex particle motion. The most common approach of analyzing the nanoparticle motion is to numerically solve the Langevin equation to analyze the Brownian trajectories^25–31^. However, solving the Langevin equation gives only one possible trajectory of the nanoparticle. Only by analyzing a large number of independent Brownian trajectories can the statistics related to the average behavior of the nanoparticles be extracted^32^. Although this approach is simple and accurate, it does have a few shortcomings. Simulating a large number of Brownian trajectories is computationally slow. In addition, the number of trajectories required to obtain acceptably accurate statistics increases with the size of the solution domain. Thus, the approach could be impractical for simulating systems with large spatial extent. The method is also geared towards calculating the statistics at a specific instance of time (usually at $t\to \infty$, when steady-state is achieved). Trying to calculate statistics at every time instance (to observe time evolution of the statistics) adds a significant amount of computational burden on top of the already slow process. The root cause of many of these issues are related to the fact that the Langevin equation calculates independent trajectories and relies on post-processing to obtain the statistics of the motion. Due to the random nature of the nanoparticle motion, one is usually more interested in the statistical behavior of the motion rather than individual trajectories. Thus, a method that directly solves the statistics can be very useful.

The Fokker-Planck equation, also known as the Kolmogorov forward equation, can solve the position probability density function (PDF) of a Brownian particle directly^33–36^. It is a partial differential equation that can be considered as an equation of motion for the distribution function of fluctuating macroscopic variables^34^. In the context of Brownian motion, the Fokker-Planck equation describes the time evolution of the position PDF. This equation can be used to predict how an initial distribution will change with time under the influence of a trapping force-field. Since the probability density is calculated directly instead of being derived from trajectories, this method can give more accurate results at a significantly less computational cost. We have found that the computational time for calculating the steady-state postion PDF of a nanoparticle in the vicinity of a single near-field trap using the Langevin equation based approach is around 35 hours. For the Fokker-Planck approach, the simulation time is approximately one hour. Also, the PDF at each time step is obtained from the Fokker-Planck analysis without any additional computation. This is especially helpful when analyzing the dynamic behavior of the system. More importantly, this approach is scalable and can be applied to systems with large spatial dimensions. Using the Langevin approach, the number of trajectories required for a specific accuracy will scale with the volume. Thus, the runtime for simulating a system with more than 2–3 traps becomes impractical. The Fokker-Planck approach imposes no such constraints. Its accuracy is dependent on the numerical grid spacing. Well established numerical methods can be used to handle large grids efficiently. Thus, analyzing complex LOC systems using the Fokker-Planck method is more practical than doing the same using the Langevin equation. To the best of the authors’ knowledge, very few works have reported the use of the Fokker-Planck equation to analyze optical traps^37^ and none have reported its use in analyzing near-field traps. Although there are works that report the motion of nanoparticles in optical traps, they mostly use the Langevin equation (or a modified version of it) for the analysis^25–29^. In this work, we report how the Fokker-Planck equation can be used to show the trapping dynamics. The presented form of the Fokker-Planck equation takes into account the spatially varying diffusion tensor which is necessary to model the hydrodynamic interactions^38–40^. We show how the time evolution data can give additional insights that can be useful for designing LOC systems. We also provide experimental data that show good agreement with the steady-state results of the Fokker-Planck analysis.

In this work, we apply the Fokker-Planck analysis on a plasmonic trap consisting of a C-shaped engraving (CSE) on a gold film. This is a well known structure that has been successfully used in many nanophotonic applications including optical trapping^18,19,41–44^. Its polarization dependent excitation and the ability to focus light to a very small spot size (<*λ*/10) makes it a desirable geometry. Spherical polystyrene beads are used as the nanoparticles. This is a common choice for many optical trapping applications^45,46^. It should be mentioned that the proposed Fokker-Planck analysis is general and would hold for any geometry and particle material. The optical force generated by a another geometry and/or particle material will be have a different spatial distribution. However, the equations and approaches used in this paper will remain unchanged. Only the appropriate force-field and the material parameters have to be plugged in.

The paper is organized as follows: first, the geometry and the coordinate system are introduced. It is followed by the calculation of the optical force field. Then the mathematical formulations related to the Fokker-Planck equation is presented. The results are given next. Discussion and concluding remarks are made on the same section. The experimental methods are discussed in brief at the end of the paper.

## Geometry

**Figure 1:**
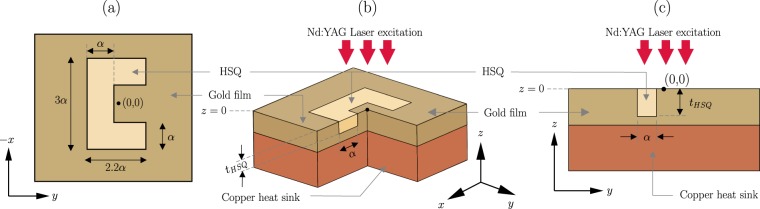
Geometry of the C-shaped engraving (**a**) Top view, (**b**) Cutaway view, (**c**) $x=0$ plane view. The coordinate system used through out this paper is also shown.

A CSE on a gold film is considered as the plasmonic near-field optical trap. The geometry of the structure is shown in Fig. 1. The engraving is filled with Hydrogen silsesquioxane (HSQ). This is a useful by-product of the fabrication process^18^. The HSQ filling creates a planar trapping surface which helps to avoid the accidental sticking of nanoparticles at the edge of the engraving. The trap operates in the reflection mode. Excitation is provided from the top in form of laser illumination (propagating along −*z* direction). A 1064 nm Nd:YAG laser is used for this purpose. A layer of water (containing nanopartilces) is placed on top of the structure. The geometrical and material parameters are listed in Table 1. The geometrical parameters are selected such that the maximum field intensity enhancement is achieved at the excitation wavelength for the given optical parameters. The dynamic viscosity parameter, $\eta$, affects the motion of the nanoparticle in water. The value of $\eta$ is taken from literature^47^ (assuming water at 300 K temperature).

**Table 1:** Geometrical and material parameters.

**CSE characteristic parameter, α**	**60 nm**
Engraving depth, *t*_*HSQ*_	150 nm
Nanoparticle radius, *r*_*o*_	150 nm
Refractive index of water, *n*_*w*_	1.33
Refractive index of HSQ, *n*_*HSQ*_	1.4
Refractive index of polystyrene, *n*_*p*_	1.58
Dynamic viscosity of water, *η*	0.8 × 10^−3^ Pa · s

A colloidal solution of nanoparticles in water is placed on top of this structure for the trapping experiment. Polystyrene beads of radius 150 nm are considered as the nanoparticles throughout this paper. Polystyrene beads are widely used in bio-applications^45,46^. However, the presented analysis in this paper is general and the same approach can be applied to model the forces on any dielectric micro/nanoparticles by using the appropriate material parameters.

## Force Calculation

The force generated by a plasmonic trap must be calculated to model the motion of a nanoparticle near it. The force-field depends on the optical response of the plasmonic structure as well as the geometrical and material properties of the nanoparticle and the surrounding medium (water). Once the electric field distribution around the structure is calculated, the Maxwell Stress Tensor (MST) method can be used to calculate the force on a nanoparticle.

**Figure 2:**
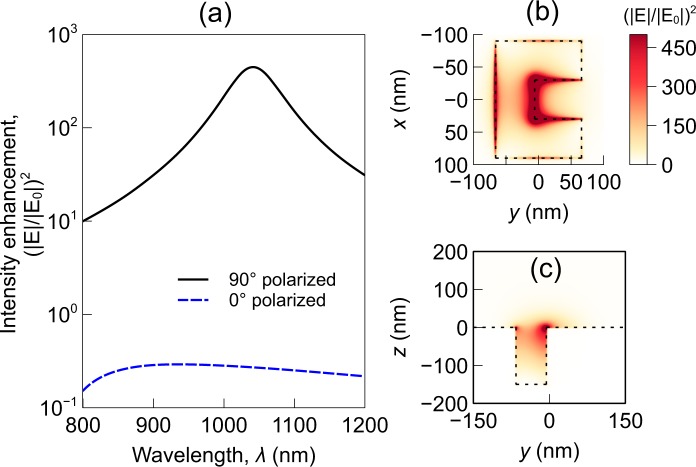
Optical intensity enhancement near a CSE (**a**) as a function of wavelength, (**b**) at $z=0$ plane, and (**c**) at $x=0$ plane. Input excitation is assumed to be 1 mW/*μ*m^2^ for all cases. The polarization angles mentioned in (**a**) is measured with respect to the $x$ axis. 1064 nm $y$-polarized (90° polarized) incident light is assumed for (**b**) and (**c**).

The optical response of the CSE is calculated using a commercial finite element solver (Comsol Multiphysics™). At first, the frequency response and the polarization response of the structure is simulated. From this simulation, the appropriate wavelength and polarization of the excitation light can be calculated. As this is done to characterize the plasmonic structure only, no nanoparticle is assumed to be present (the presence of the nanoparticle is considered for all subsequent simulations related to the optical force calculations). The optical response of structure is shown in Fig. 2. The incident light intensity is assumed to be 1 mW/*μ*m^2^ throughout this paper. This is a commonly used intensity value for near-field optical trapping^9,12^ The laser illumination is modeled as a Gaussian beam. The surrounding medium is assumed to be water. The structure is found to be resonant at *y*-polarized light of wavelength near 1064 nm. The polarization dependency is due to the asymmetrical structure of the CSE^41^. Figure 2 shows that the CSE can produce a very high localized intensity enhancement. Also, the high intensity region is focused on a very small volume^41,48^. The spot size is much smaller than the nanoparticle size considered in this paper. The small spot size creates a high intensity gradient which in turn produces a strong trapping force.

For calculating the force, the nanoparticle must be taken into account when calculating the field distribution. Once the electromagnetic fields are calculated for a given position of the nanoparticle, the force can be obtained by the MST method^49,50^:

1 $$\overleftrightarrow{{\bf{T}}}={\varepsilon }_{w}({\bf{E}}{\bf{E}}-\frac{1}{2}|{\bf{E}}{|}^{2}\overleftrightarrow{{\bf{I}}})+{\mu }_{w}({\bf{H}}{\bf{H}}-\frac{1}{2}|{\bf{H}}{|}^{2}\overleftrightarrow{{\bf{I}}}),$$

2 $${\langle {\bf{F}}\rangle }_{t}=\mathop{\int }\limits_{S}\,{\langle \overleftrightarrow{{\bf{T}}}\rangle }_{t}\cdot \hat{{\bf{n}}}\,{\rm{d}}S.$$

**Figure 3:**
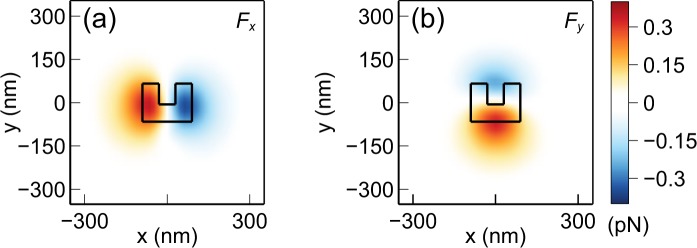
Force distribution in the $z=155\,{\rm{nm}}$ plane (bottom of the nanoparticle is 5 nm above the plasmonic surface) near the CSE (**a**) $x$ component of the force, (**b**) $y$ component of the force.

**Figure 4:**
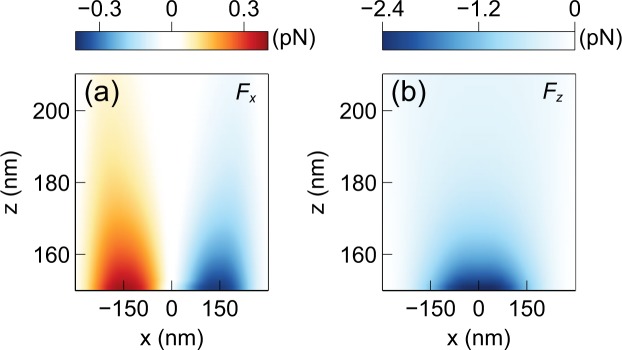
Force distribution in the $y=0$-plane near the CSE (**a**) $x$ component of the force, (**b**) $z$ component of the force.

Here $\overleftrightarrow{{\bf{T}}}$ is the Maxwell stress tensor, ${\bf{E}}$ is the electric field, ${\bf{H}}$ is the magnetic field, ${\varepsilon }_{w}$ and ${\mu }_{w}$ are the permittivity and permeability of the surrounding medium (water), respectively, $\overleftrightarrow{{\bf{I}}}$ is the identity tensor, ${\bf{F}}$ is the net electromagnetic force acting on the particle, *S* is the outer surface of the nanoparticle, and $\hat{{\bf{n}}}$ is the surface normal to $S$. ${\langle \cdot \rangle }_{t}$ represents the time-averaged value. This calculation gives the force value at the given position of the nanoparticle. To obtain the force value at a different point in space, the nanoparticle must be placed at that point and the fields and the force must be recalculated. Although this process is cumbersome, it gives very accurate results. To calculate the force-field, the nanoparticle position is swept in a discrete three dimensional grid and the entire process is repeated to generate a discrete dataset of force values. A non-uniform grid with spacing varying from $0.1{r}_{o}$ to $1.1{r}_{o}$ is used, where ${r}_{o}=150\,{\rm{nm}}$ is the radius of the nanoparticle. This range of grid size is optimum and it was determined by trial and error. Small grid sizes are used near the center of the structure as that region has the highest intensity gradient and requires the most resolution. Three dimensional interpolation can be applied on this dataset to calculate the force in any point. The calculated force-field is shown in Figs 3 and 4. It can be seen that pico-newton level forces are generated that pull the nanoparticle towards the center of the trap. It is noted that ${F}_{z}$ has a higher peak value than ${F}_{x}$ and ${F}_{y}$. This is a result of the larger field intensity gradient along the $z$ direction compared to the $x$ and $y$ directions. This occurs because of the physical extent of the CSE in $xy$ plane. The field intensity has a nonzero value over the entire CSE surface parallel to the $xy$ plane. However, along the $z$ direction the intensity drops to almost zero very rapidly due to the evanescent nature of the fields. For the same reason, the fore range (and therefore the trapping range) is shorter along the $z$ direction compared to the $x$ and $y$ directions^32^. The obtained force-field will be used to model the nanoparticle motion in the subsequent sections.

## Fokker-Planck Analysis

The Fokker-Planck equation provides a self-consistent description of the time evolution of the position PDF. Given an arbitrary initial position PDF, the equation can be used to model how that PDF changes with time. It takes into account the diffusion mechanism as well as the drift motion induced by an external force. In the context of a near-field optical trap, the drift motion would be caused by the optical trapping force and the diffusion process would represent the Brownian motion of a nanoparticle in the suspension medium. A nanoparticle suspended in water near an optical trap experiences drift motion due to the trapping force as well as Brownian motion due to the random collisions with the water molecules. Thus the equation is well suited to model the particle dynamics near an optical trap. For time independent drift and diffusion coefficients, the Fokker-Planck equation is given by^33,34^:

3 $$\frac{\partial P({\bf{r}},t)}{\partial t}=-\,\sum _{i=1}^{3}\,\frac{\partial }{\partial {r}_{i}}({A}_{i}({\bf{r}})P({\bf{r}},t))+\frac{1}{2}(\sum _{i=1}^{3}\,\sum _{j=1}^{3}\,\frac{{\partial }^{2}}{\partial {r}_{i}\partial {r}_{j}}({B}_{ij}^{2}({\bf{r}})P({\bf{r}},t))){\rm{.}}$$

Here ${\bf{r}}$ is the position of the center of the nanoparticle, *r*_1_, *r*_2_ and *r*_3_ corresponds to the Cartesian $x$, $y$ and $z$ directions respectively, and $P({\bf{r}},t)$ is the position PDF at time $T$. ${A}_{i}$ terms determine how the external force (optical force) effects the particle motion (drift mechanism). ${B}_{ij}$ terms characterize the Brownian dynamics. The terms ${A}_{i}$ are related to the drift coefficient (the relationship is approximately linear) and the terms ${B}_{ij}$ are are proportional to the square root of the diffusion coefficient. The exact relationships among the parameters will be established later in this section. The drift coefficient and the diffusion coefficient determine how the material properties of the system (nanoparticle and the suspension medium) effects the drift and diffusion mechanisms, respectively. We assume these coefficients to be time independent as the material properties of the system are not expected to vary with time. In general, these coefficients are expressed in terms of tensors. For a diagonal diffusion tensor, Eq. 3 can be simplified as:

4 $$\frac{\partial P({\bf{r}},t)}{\partial t}=-\,\sum _{i=1}^{3}\,\frac{\partial }{\partial {r}_{i}}({A}_{i}({\bf{r}})P({\bf{r}},t))+\frac{1}{2}(\sum _{i=1}^{3}\,\frac{{\partial }^{2}}{\partial {r}_{i}^{2}}({B}_{ii}^{2}({\bf{r}})P({\bf{r}},t))){\rm{.}}$$

The equation can be rewritten in a more convenient form as:

5 $$\frac{\partial P({\bf{r}},t)}{\partial t}=({ {\mathcal L} }_{x}+{ {\mathcal L} }_{y}+{ {\mathcal L} }_{z})P({\bf{r}},t),$$

where the operators ${ {\mathcal L} }_{i}$ are defined as:

6 $${ {\mathcal L} }_{i}=-\,\frac{\partial {A}_{i}({\bf{r}})}{\partial {r}_{i}}+\frac{1}{2}\frac{\partial {B}_{ii}^{2}({\bf{r}})}{\partial {r}_{i}^{2}}{\rm{.}}$$

Note that assuming the diffusion tensor to be diagonal is justified for this case as any physical axis of the system can be aligned with the simulation/experimental reference frame.

The terms ${A}_{i}$ and ${B}_{ii}$ are related to various system parameters such as the force-field and the diffusion tensor. The relationships between these quantities must be established to convert the general form of the above equations to a form more suitable for the near-field trapping problem under consideration. It is easier to do so by starting with the Langevin equation. There is an underlying stochastic Langevin equation that corresponds to a Fokker-Planck equation^51^. To relate the terms of the Fokker-Planck equation with the physical parameters of the system, we first relate them to the terms of that Langevin equation. For a nanoparticle suspended in a low Reynolds number fluid, the Brownian motion of the particle can be expressed using the modified Langevin equation^25,26^:

7 $$\dot{{\bf{r}}}(t)=\frac{\overleftrightarrow{{\bf{D}}}({\bf{r}})}{{k}_{B}T}{\bf{F}}({\bf{r}})+\sqrt{2}{\overleftrightarrow{{\bf{D}}}}_{1/2}({\bf{r}}){\bf{W}}(t).$$

Here, ${\bf{F}}({\bf{r}})$ is the optical trapping force-field, ${k}_{B}$ is the Boltzmann constant, $T$ is the temperature, $\overleftrightarrow{{\bf{D}}}$ is the diffusion tensor, and ${\bf{W}}(t)$ is a vector white noise term. The tensor ${\overleftrightarrow{{\bf{D}}}}_{1/2}$ is obtained by taking element-wise square root of $\overleftrightarrow{{\bf{D}}}$. Each Cartesian component of ${\bf{W}}(t)$ is a Gaussian random process with zero mean and unit variance. $\overleftrightarrow{{\bf{D}}}$ is a diagonal tensor with ${D}_{11}({\bf{r}})={D}_{22}({\bf{r}})={{\bf{D}}}_{\parallel }({\bf{r}})$ and ${D}_{33}({\bf{r}})={D}_{z}({\bf{r}})={D}_{\perp }({\bf{r}})$. The components are defined as:

8 $${D}_{\parallel }({\bf{r}})=\frac{{k}_{B}T}{6\pi \eta {r}_{o}}(1-\frac{9{r}_{o}}{16z}+\frac{{r}_{o}^{3}}{8{z}^{3}}-\frac{45{r}_{o}^{4}}{256{z}^{4}}-\frac{{r}_{o}^{5}}{16{z}^{5}}),$$

9 $${D}_{\perp }({\bf{r}})=\frac{{k}_{B}T}{6\pi \eta {r}_{o}}(\frac{6{z}^{2}+2{r}_{o}z}{6{z}^{2}+9{r}_{o}z+2{r}_{o}^{2}}){\rm{.}}$$

Here $\eta$ is the dynamic viscosity of water, ${r}_{o}$ is the radius of the nanoparticle, and $z$ is the $z$-coordinate of the center of the nanoparticle. The dependency on $z$ arises from the hydrodynamic interactions^38–40^ between the nanoparticle and the water layers near the solid surface located at $z=0$ plane. Both ${D}_{\parallel }({\bf{r}})$ and ${D}_{\perp }({\bf{r}})$ approach the isotropic value of $\frac{{k}_{B}T}{6\pi \eta {r}_{o}}$ as the separation between the solid surface and the particle increases. Also note that the temperature is assumed to be uniform through out the simulation domain ($T$ is not dependent on ${\bf{r}}$). As the trapping structure employs a heat sink, we have found that the temperature rise is negligible and has very little spatial dependence^18^. Thus assuming a uniform temperature profile is reasonable.

As the terms of the Langevin equation have been defined in terms of the physical quantities of the system, now we can relate them to the terms of the Fokker-Planck equation. For the current case, it can be shown that they are related by the following equations^34,52^:

10 $${A}_{i}({\bf{r}})=\frac{{D}_{ii}({\bf{r}})}{{k}_{B}T}{F}_{i}({\bf{r}})+{D}_{1/2,ii}({\bf{r}})\frac{\partial }{\partial {r}_{i}}({D}_{\frac{1}{2},ii}({\bf{r}})),$$

11 $${B}_{ii}({\bf{r}})=\sqrt{2}{D}_{1/2,ii}({\bf{r}}).$$

where $i=1,2,3$ represents the Cartesian $x$, $y$, and $z$ components. For diffusion tensors that do not vary with space, the equations reduce to the simpler forms used in^37^.

All the terms of Eq. 4 have been related to the optical force and the physical parameters of the system. Now we focus on solving the equation. To solve the partial differential equation in a domain ${\rm{\Omega }}$ with boundary $\partial {\rm{\Omega }}$, the boundary conditions must be defined. We assume the system to be closed and enforce a no-flux boundary condition^34,53^. In other words, we assume that the probability current, ${\bf{J}}$, at the boundaries is zero as the nanoparticle cannot leave the system. So, the boundary conditions are:

12 $${J}_{i}({\bf{r}},t){|}_{{\bf{r}}=\partial {\rm{\Omega }}}=0$$

where

13 $${J}_{i}({\bf{r}},t)={A}_{i}({\bf{r}})P({\bf{r}},t)-\frac{1}{2}\frac{{\rm{\partial }}}{{\rm{\partial }}{r}_{i}}({B}_{ii}^{2}({\bf{r}})P({\bf{r}},t)).$$

The no-flux boundary condition can be justified if a sufficiently large region around the trap is selected as the domain (${\rm{\Omega }}$) where the nanoparticle can be assumed to be bounded. The solid reflective surface at $z=0$ is also consistent with this boundary condition. We assume that a nanoparticle hitting this solid surface would bounce off without any loss of energy and follow the mechanics of elastic collisions. In the presence of a trapping force-field, it would be reasonable to assume that the nanoparticle would remain in ${\rm{\Omega }}$ (for a sufficiently large ${\rm{\Omega }}$) as its position evolves with time. For this assumption to be valid, we have found that the size of the simulation domain should be set such that the radial distance from the initial PDF to a non-physical simulation boundary is at least as large as the radial distance from the initial PDF to the trap center. However, the required simulation domain size depends on the magnitude of the optical force, material properties etc. and would not necessarily follow this rule in general.

The Fokker-Planck equation can be solved numerically on a discrete space-time finite difference grid using the Douglas alternate direction implicit (ADI) scheme^54,55^. Equation 5 can be written in terms of discrete finite difference operators and converted into the following difference equations using the Douglas method^54^:

14 $$({{\rm{\Delta }}}_{x}-\frac{2}{{\rm{\Delta }}t}){P}_{n+1}^{\ast }=-\,({{\rm{\Delta }}}_{x}+2{{\rm{\Delta }}}_{y}+2{{\rm{\Delta }}}_{z}+\frac{2}{{\rm{\Delta }}t}){P}_{n},$$

15 $$({{\rm{\Delta }}}_{y}-\frac{2}{{\rm{\Delta }}t}){P}_{n+1}^{\ast \ast }=-\,({{\rm{\Delta }}}_{x}+{{\rm{\Delta }}}_{y}+2{{\rm{\Delta }}}_{z}+\frac{2}{{\rm{\Delta }}t}){P}_{n}-{{\rm{\Delta }}}_{x}{P}_{n+1}^{\ast },$$

16 $$({{\rm{\Delta }}}_{z}-\frac{2}{{\rm{\Delta }}t}){P}_{n+1}=-\,({{\rm{\Delta }}}_{x}+{{\rm{\Delta }}}_{y}+{{\rm{\Delta }}}_{z}+\frac{2}{{\rm{\Delta }}t}){P}_{n}-{{\rm{\Delta }}}_{x}{P}_{n+1}^{\ast }-{{\rm{\Delta }}}_{y}{P}_{n+1}^{\ast \ast }{\rm{.}}$$

Here Δ*t* is the discrete time step, ${{\rm{\Delta }}}_{x}$, ${{\rm{\Delta }}}_{y}$ and ${{\rm{\Delta }}}_{z}$ are the discrete finite difference operators^56,57^ corresponding to the continuous operators ${ {\mathcal L} }_{x}$, ${ {\mathcal L} }_{y}$ and ${ {\mathcal L} }_{z}$, respectively, the subscript *n* indicates time index ($n=0,1,2\ldots$), and ${P}_{n}$ is the position PDF at time index ${t}_{n}=n{\rm{\Delta }}t$. ${P}_{n+1}^{\ast }$ and ${P}_{n+1}^{\ast \ast }$ are intermediate solutions. Using these difference equations, the position PDF at any time can be calculated from an arbitrary initial distribution ${P}_{0}$.

## Results and Discussion

**Figure 5:**
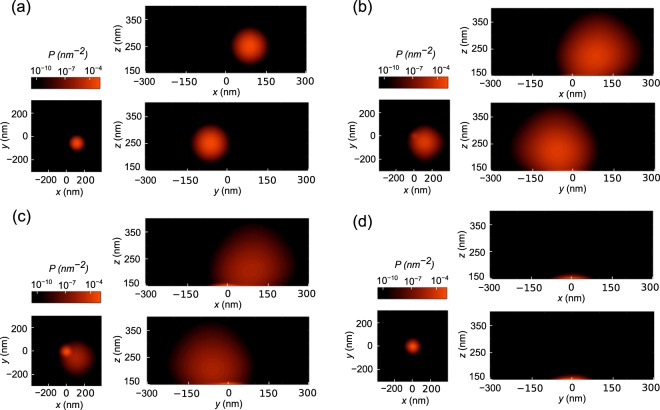
The marginal PDF of the nanoparticle position at: (**a**) $t=0\,\mu {\rm{s}}$, (**b**) $t=280\,\mu {\rm{s}}$, (**c**) $t=424\,\mu {\rm{s}}$, and (**d**) $t=744\,\mu {\rm{s}}$.

The Fokker-Planck equation is numerically solved to calculate the position distribution of a nanoparticle near a CSE. The temperature is assumed to be $T=300\,{\rm{K}}$, which is consistent with the experimental conditions. The dynamic viscosity of water is taken to be, $\eta =0.8\times {10}^{-3}\,{\rm{Pa}}\cdot {\rm{s}}$^47^. The initial PDF is selected as a Gaussian centered around the point $(90\,{\rm{nm}},-\,60\,{\rm{nm}},250\,{\rm{nm}})$ with a variance of 200nm^2^. It should be noted that this choice is arbitrary. Identical steady-state distributions were achieved for other initial distributions as long as they were located within the capturing range of the trap^32^ (a few such cases are included in the supplementary materials). The time step was taken to be ${\rm{\Delta }}t=4{\rm{\mu }}{\rm{s}}$. The evolution of the position PDF is shown in Fig. 5. Since the three dimensional PDF is difficult to visualize, the marginal densities at $xy$, $yz$ and $xz$ planes are shown here. A video file is included in the supplementary materials (c60_150 nm_initial(90,−60,250)_var200.gif) to show the time evolution. The supplementary files also include videos for other initial distributions. The plots show that the position PDF moves toward the trap center with time. Initially, the variance of the PDF increases as the distribution spreads out in space. As parts of the distribution get near the trap, the optical force starts taking effect and the distribution is slowly pulled near the trap center. During this phase, the variance of the PDF starts to decrease as the position PDF converges to its steady-state form. It can be noted that the motion along the $z$ direction is initially faster than the motion along $x$ and $y$ directions. This is due to the fact that the force along the $z$ direction, ${F}_{z}$, is stronger than the force in other directions. The $x$ and $y$ components of the force (${F}_{x}$ and ${F}_{y}$, respectively) have more impact once the nanoparticle is pulled near the solid surface at $z=0$.

**Figure 6:**
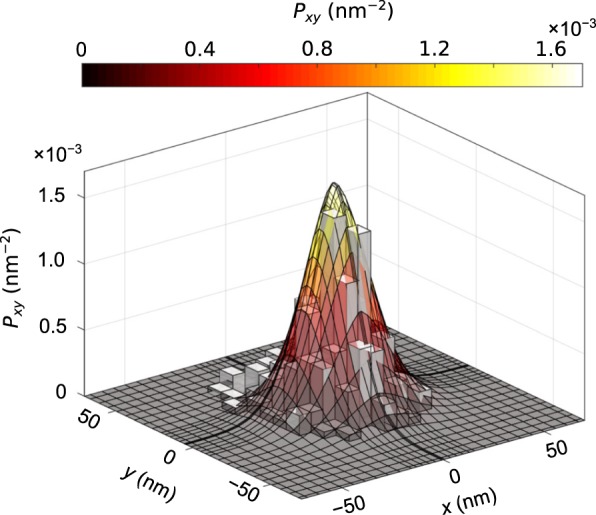
Steady-state marginal probability distribution along the $xy$ plane. The surface plot is obtained from the Fokker-Planck equation. The histogram represents experimental data.

The probability density converges to a steady-state as $t\to \infty$. The numerical results show that a reasonable approximate for the steady-state distribution can be obtained for time $t > 550\,\mu {\rm{s}}$. The results were calculated for a longer duration to verify the convergence of the statistics. The steady-state statistics were collected at $t=750\,\mu {\rm{s}}$. For comparison, we can calculate the diffusion time of a nanoparticle traveling the same distance (from the location of the initial PDF to the trap center) in absence of any external force. The mean-squared displacement (MSD) for such a case can be approximated as 6*Dt*^58^. Setting the MSD equal to the square of travel distance, we find the diffusion time to be 7222 *μ*s. Note that this is a rough estimate assuming 3D isotropic diffusion. The required time is expected to be much smaller when the nanoparticle is under the influence of an optical trapping force. So, the reduction in the required diffusion time by an order of magnitude is reasonable. This time scale is also consistent with the experimental observations. The steady-state marginal distribution along the $xy$ plane is shown in Fig. 6. The experimental position distribution data is also shown in the same plot as a histogram. The experiment is performed under the same conditions and with the same materials as considered in the simulations. The experimental data of particle position is obtained by analyzing the video of a trapping event. The experimental setup is discussed in the experimental methods section. Figure 6 shows that the steady-state nanoparticle position PDF obtained from the Fokker-Planck equation is in good agreement with the experimental data.

**Figure 7:**
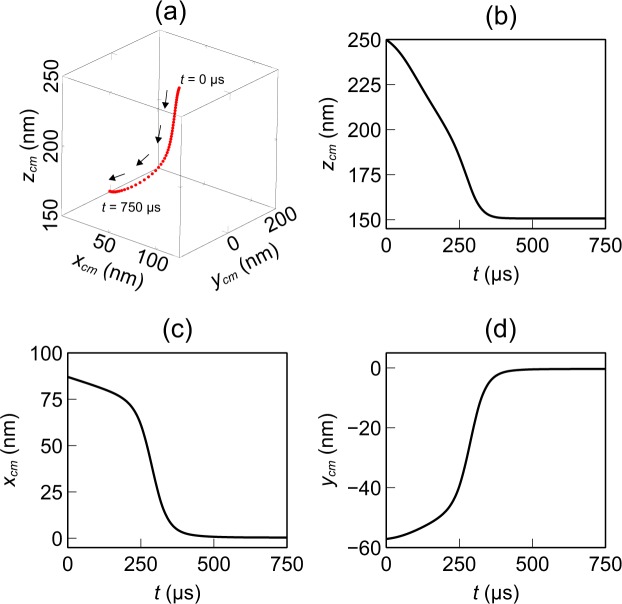
The average trajectory of a nanoparticle near a CSE: (**a**) 3D scatter plot, (**b**) $z$-coordinate, (**c**) $x$-coordinate, and (**d**) $y$-coordinate of the center of mass of the position PDF. The flattening of the curves indicate steady-state condition.

Unlike the Langevin equation, the Fokker-Planck equation does not calculate any single trajectory of the nanoparticle. It calculates the position probability density directly, which is often the desired data. However, for some applications, calculating a trajectory can be useful. It is possible to estimate some trajectory related information from the position PDF data. For example, an *average trajectory* can be defined from the time series probability density data. For each time instance, the center of mass of the position PDF, $({x}_{cm},{y}_{cm},{z}_{cm})$, can be calculated using the following equation:

17 $${x}_{cm}({t}_{n})=\frac{1}{\sum _{i,j,k}\,P({x}_{i},{y}_{j},{z}_{k},{t}_{n})}\sum _{i,j,k}\,P({x}_{i},{y}_{j},{z}_{k},{t}_{n}){x}_{i}({t}_{n}),$$

where $({x}_{i},{y}_{j},{z}_{k})$ are the finite difference grid points where the Fokker-Planck equation was solved, and ${x}_{cm}({t}_{n})$ is the $x$-coordinate of the center of mass of the position PDF at time ${t}_{n}$. Similar expressions can be used to calculate ${y}_{cm}({t}_{n})$ and ${z}_{cm}({t}_{n})$. The path that the center of mass of the position PDF traces out with time can be considered as the average trajectory of the nanoparticle. This trajectory is shown in Fig. 7. The convergence of the solution at *t* ≈ 500 *μ*s can be observed from these plots as well. The plots also show that initially ($t < 250\,\mu {\rm{s}}$) the motion is predominantly along the −*z* direction. As explained before, this can be attributed to the higher magnitude of ${F}_{z}$ compared to ${F}_{x}$ and ${F}_{y}$. Once the nanoparticle reaches the bottom surface of the structure (when particle $z$ coordinate is around 150 nm), no further motion along $z$ direction is possible. Also, the nanoparticle experiences stronger ${F}_{x}$ and ${F}_{y}$ at that time and a major portion of the motion along $xy$ plane occurs after $t\approx 250\,\mu {\rm{s}}$ instance. This insight into the trapping dynamics can be useful when designing LOC systems.

The steady-state position PDF obtained from the Fokker-Planck method is consistent with experimental results. Unlike the trajectory based approach that uses the Langevin equation, this method solves the position PDF directly. As a result, for the same target accuracy, the computational cost of the proposed approach is significantly less. In addition, using the numerically efficient Douglas ADI solver further reduces the simulation time. It was found that the computational time for the Fokker-Planck approach was approximately 56 minutes whereas the computational time for evaluating Brownian trajectories from the Langevin equation required 35 hours (for 300,000 Langevin trajectories). The run time for calculating the steady-state PDF from the initial PDF is considered here. The computation was done using MATLAB in a machine with Intel®Xeon®E5-2690 v3 2.6 GHz CPU and 256GB RAM. The proposed approach is 35 times faster which is a significant improvement. Note that the lowest probability value that can be calculated from 300,000 Langevin trajectories is 1/300,000 per volume element. The number 300,000 was selected based on trial and error. The number of trajectories were varied until the Langevin approach produced statistics with similar levels of accuracy as the Fokker-Planck method. The grid spacing, time step, and the simulation duration were kept the same for both methods^26^. The solid surfaces were modeled as perfectly reflecting (nanoparticles would experience elastic collisions with the solid surfaces) for both cases. For a given grid size, increasing the statistical accuracy (or resolution) of the Langevin approach involves increasing the number of trajectories. On the other hand, for a given grid size, the statistical accuracy of the Fokker-Planck approach is limited by the numerical errors only.

In addition to the faster run time, perhaps a bigger advantage of the Fokker-Planck approach is that it can be applied to a larger system. Consider a nanoparticle in a LOC system containing multiple trapping sites. The region of interest where the nanoparticle can move is much larger in this case than the case of an isolated trap. If the Langevin equation is used for such systems, a very large number of trajectories need to be calculated to analyze the statistical properties. This is due to the fact that the accuracy of the statistics depend on the density of the trajectories. The position PDF at a given point is calculated by counting how many trajectories end up in a small volume element around that point^32^. Increasing the volume decreases the expected number of trajectories at a given spatial volume. Therefore, some regions of the solution domain will have too few trajectories to calculate a reliable value of the PDF. Hence, for a larger volume, the number of trajectories required for the same level of accuracy goes up. This can increase the computational cost significantly. Note that this cost is on top of the computational cost associated with simulating a larger grid. This restricts the usability of the method for large systems. On the other hand, as the Fokker-Planck approach solves the statistics directly, it does not suffer from the same shortcoming. It can be argued that the Fokker-Planck approach would require a larger finite difference grid for a larger system. However, that comparison is not completely accurate. A larger grid size implies that the statistics would be calculated at more points. The accuracy of the statistics at each point in the grid is independent of the actual number of grid points (for a reasonably well defined grid). Whereas for the Langevin equation, the accuracy of the statistics depends on how many trajectories pass through the point (or through a small region near the point) under consideration. In addition, non-uniform grids and multi-grid methods can be used to tackle large grids. Other issues related to a large system matrix can be addressed using well known numerical techniques^56,57^. Thus, the Fokker-Planck based method is well-suited for tackling LOC systems.

We have presented an alternative method to model motion of Brownian nanoparticles in an optical force-field that can be scaled for large systems. The proposed analysis is general and can be applied to any geometry. Since no work has been reported in the literature (to the best of our knowledge) related to the Fokker-Planck equation applied to near-field traps, this paper can be of interest to the optical trapping community.

## Experimental Methods

The CSE is fabricated using the same method described in^18^. The dimensions of the fabricated structure are consistent with the simulation parameters. An SEM image of the CSE is included in the supplementary materials. The experiment is done using a Nikon TE2000-U inverted microscope. Fluorescent polystyrence beads (from Thermo Fisher Scientific™) are used as the nanoparticles. A dilute aqueous solution of nanoparticles is placed on a 0.17 mm thick glass coverslip. The suspension medium is water, which is consistent with our simulation parameters. The fabricated chip with the CSE is placed upside down on top of the glass slide. The setup is placed on a picromotor stage on the microscope. The picomotor stage is moved so that the CSE is centered. Nd:YAG laser excitation of wavelength 1064 nm is provided to the chip through a Nikon CFI Plan Fluor 40X objective lens (NA = 0.75) to excite the CSE. The loosely focused laser creates a Gaussian beam at the sample plane. A half-wave plate on a rotary stage is used to control the polarization of the laser. The half-wave plate is rotated so that the polarization of the laser is aligned with the orientation of the CSE (that is, when the beam polarization is along the $y$ direction on the sample plane). An initial calibration of the experimental setup is performed to relate the angular position of the half-wave plate to the polarization of the laser beam on the sample plane. A complete ray-path of the optical setup is provided in the supplementary materials.

A sCMOS camera (PCO Edge™5.5) is used to record the trapping event. A particle tracking code (based on the IDL tracking algorithm) is used to track the position of the trapped nanoparticle. From the position information, the experimental probability density is calculated.

## Supplementary information

Supplementary information

Video file showing time evolution of PDF

Video file showing time evolution of PDF

Video file showing time evolution of PDF

Video file showing time evolution of PDF

## Data Availability

The datasets generated and/or analyzed during the current study are available from the corresponding author on reasonable request.
