# Supplementary material for: Fokker-Planck analysis of optical near-field traps
Source: Sci Rep. 2019 Jul 2;9:9557. doi: 10.1038/s41598-019-45609-x (PMC6606609; doi:10.1038/s41598-019-45609-x)
Supplement: Supplementary file 1 — Supplementary information [file 41598_2019_45609_MOESM1_ESM.pdf]

Supplementary Information: Fokker-Planck analysis of optical near-field traps

Mohammad Asif Zaman[1,*], **Punnag Padhy**[1], **and Lambertus Hesselink**[1]

[1]Stanford University, Electrical Engineering, Stanford, CA 94305, USA
[*]zaman@stanford.edu

ABSTRACT

Additional details about the methods and procedures related to the study are reported in this document.

Hydrodynamic Interactions

The Brownian motion of a nanoparticle is affected by the presence of solid surfaces in its vicinity. The hydrodynamic interactions between the solid surface and the suspending fluid influences the Brownian motion of the nanoparticles. For near-field traps, the location of a trapped nanoparticle is close to the surface. As a result, they hydrodynamic interactions alter its diffusive properties. In addition, since it interacts with the solid surface in only one direction, an anisotropic behavior is created. This can be mathematically modeled using a diffusion tensor (instead of using a constant diffusion coefficient). The equations in the paper describe how the diffusion tensor depends on the distance between the nanoparticle and the surface. The components of the diffusion tensor are plotted as a function of z (the separation between the solid surface at $z = 0$ and the nanoparticle center) in Fig. 1. Here, $D_0 = \frac{k_B T}{6\pi\eta r_o}$ is the free space diffusion coefficient, r_o is the radius of the nanoparticle, $T = 300\,\text{K}$ is the temperature, $\eta = 0.8 \times 10^{-3}\,\text{Pa}\cdot\text{s}$ is the dynamic viscosity of water, $k_B = 1.38 \times 10^{-23}\,\text{JK}^{-1}$ is the Boltzmann constant, $D_{||}$ is the component of the diffusion tensor parallel to the solid surface (xy plane) and, $D_\perp$ is the component of the diffusion tensor along the direction perpendicular to the solid surface (z direction). It can be noted that both $D_{||}$ and $D_\perp$ approach D_0 as $z \to \infty$. This is expected as the effects of hydrodynamic interactions diminish as the separation from the solid surface increases.

Video File

The GIF files provided with the supplementary information show the time evolution of the position probability density function (PDF) of the nanoparticle. Video files are included for four different initial PDFs. The initial PDFs are Gaussian distributions with different mean and variance values. The parameters of the PDFs are listed in Table 1. The videos show how an initial arbitrary PDF evolves into the final PDF. The trapping dynamics can be visualized more clearly from the video file than from the static plots. It can be noted that any arbitrary initial PDF that is within the capturing range of the trap gives the same steady-state final PDF..

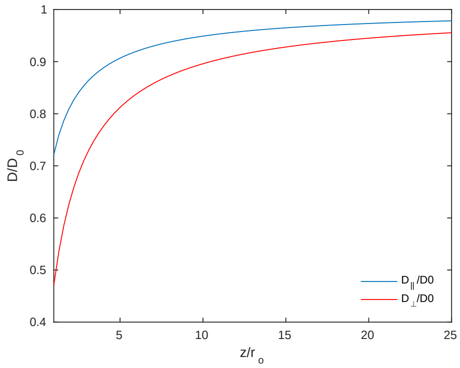

Figure 1. Components of the diffusion coefficient in the perpendicular direction and in the parallel direction.

Table 1. Initial PDFs used for making the video files

Filename	Initial PDF mean (nm)	Initial variance (nm^2)
c60_150nm_initial(90,-60,250)_var200.gif	(90,-60,250)	200
c60_150nm_initial(30,80,300)_var450	(30,80,300)	450
c60_150nm_initial(-120,-120,220)_var112	(-120,-120,220)	112.5
c60_150nm_initial(-130,100,240)_var312	(-130,100,240)	312.5

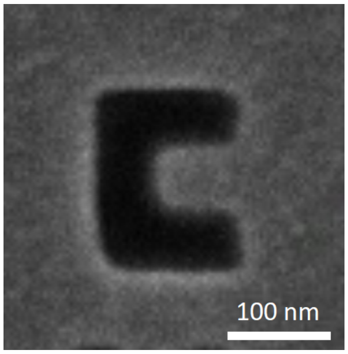

Figure 2. SEM image of a fabricated CSE. The dark regions are the HSQ filled CSE.

Sample Preparation

The SEM image of a fabricated CSE is shown in Fig. 2. The dimensions of the fabricated structure is consistent with our simulation parameters. Fluorescent polystyrene beads of radius 150 nm are considered as nanoparticles. Due to the fluorescence, they can be imaged and tracked using particle tracking algorithms despite their small size. The nanoparticles were suspended in water. Deionized water is used to dilute the concentration of the nanoparticles to approximately $10^9\,\mathrm{mL}^{-1}$. The low concentration allows us to work with a single bead at a time. A well of diameter 5 mm is created on a standard 0.17 mm glass coverslip using double sided tape. The dilute solution of nanoparticles is placed on the well using a pipette. The fabricated chip is placed on top (the side with the plasmonic structures facing the cover glass). The double sided tape holds the chip to the coverslip. This sandwiches the nanoparticle solution between the cover glass and the plasmonic chip. The sample is placed on an inverted microscope (Nikon TE2000-U) with the glass side facing down. The laser excitation coming thought the objective lens of the microscope passes through the glass and the liquid and hits the chip surface to excite the plasmonic structures.

Experimental setup

A schematic of the optical system used for the experiment is shown in Fig. 3. Light from the Nd:YAG laser goes through a polarizing beamsplitter and a half-wave plate to create a linearly polarized beam. The half-wave plate is mounted on a rotary stage. By rotating the half-wave plate it is possible to control the polarization angle of the laser beam. This beam is directed toward the objective lens (Nikon Plan Fluor 40X, NA = 0.75) by a dichroic mirror. A variable beam contractor was used to reduce the beam diameter entering the objective lens. A smaller diameter beam at the back aperture of the objective experience a reduced NA and creates a larger spot size on the sample plane. This makes it easier to illuminate the CSE with a relatively uniform illumination. The full-width half-maximum (FWHM) spot size on the sample plane was approximately $4\,\mu$m (compared to the CSE footprint of $180\,\mathrm{nm} \times 132\,\mathrm{nm}$). The sample is placed on a picomotor stage directly above the objective lens. The laser excites the plasmonic structure on the sample. The laser excitation is controlled so that the incident power density becomes $1\,\mathrm{mW}/\mu\mathrm{m}^2$. The reflected laser light is redirected towards a CCD beam imaging camera using two more dichroic mirrors.

A mercury lamp is used to excite the fluorescence. A separate white light source is used for general illumination. Light from these sources are reflected from a dichroic mirror and directed towards the sample though the objective lens. The light reflected from the sample is imaged using a CMOS camera (PCO Edge 5.5). The optical path of each of these rays are shown in Fig. 3. The filtered mercury light and the white light at the sample plane have significantly less power compared to the laser illumination. Also, the wavelength of these sources are far away from the resonance wavelength of the CSE. Thus, this light sources are expected to have no impact on the trapping dynamics.

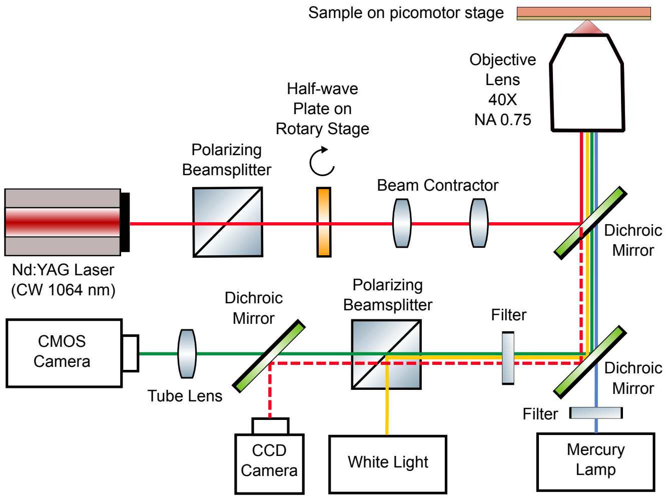

Figure 3. Schematic of the optical setup. The solid red line represents the Nd:YAG excitation ray and the dashed red line is the reflected laser ray path. Blue line is the light from the mercury lamp used for exciting the fluorescence. The green ray is the fluorescent emission from the nanoparticles.
