# Supplementary figures and images for: Fokker-Planck analysis of optical near-field traps
Source: Sci Rep. 2019 Jul 2;9:9557. doi: 10.1038/s41598-019-45609-x (PMC6606609; doi:10.1038/s41598-019-45609-x)

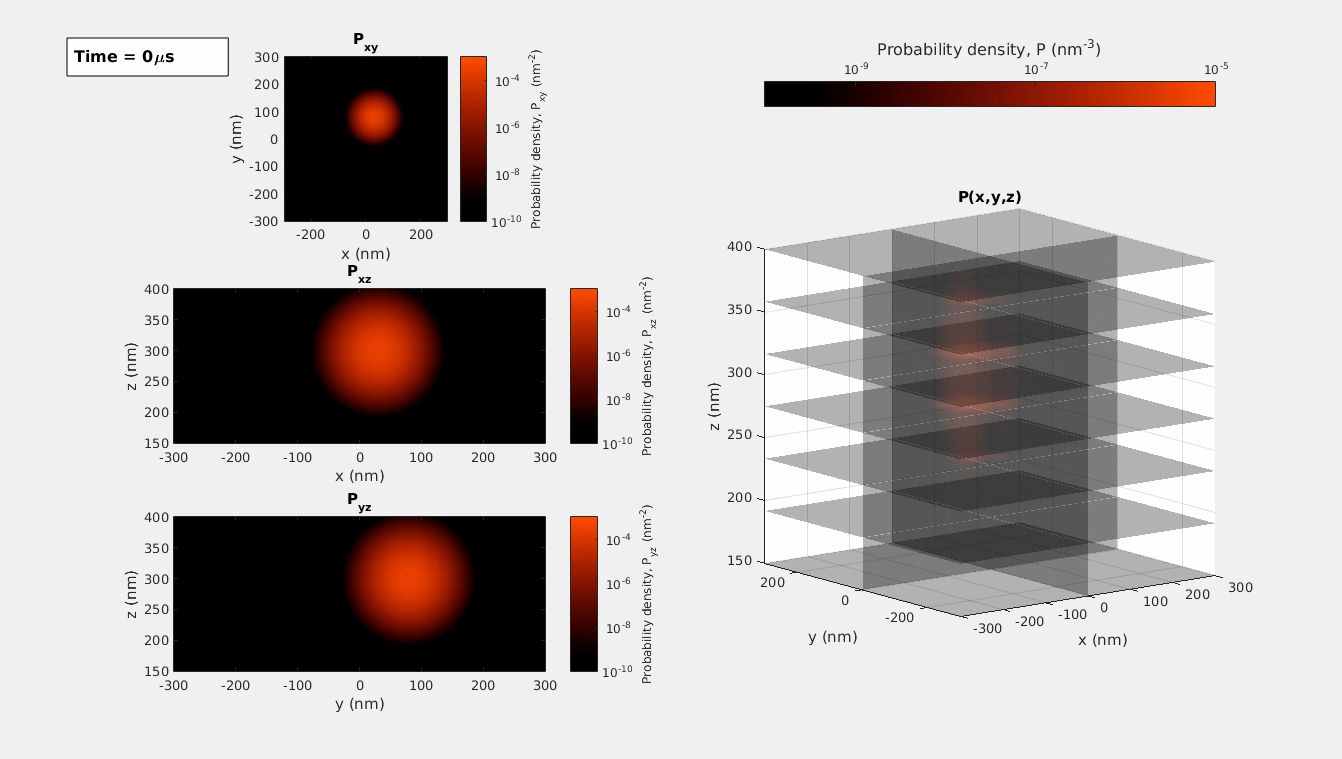

Supplement: Supplementary file 2 — Video file showing time evolution of PDF [file 41598_2019_45609_MOESM2_ESM.gif]

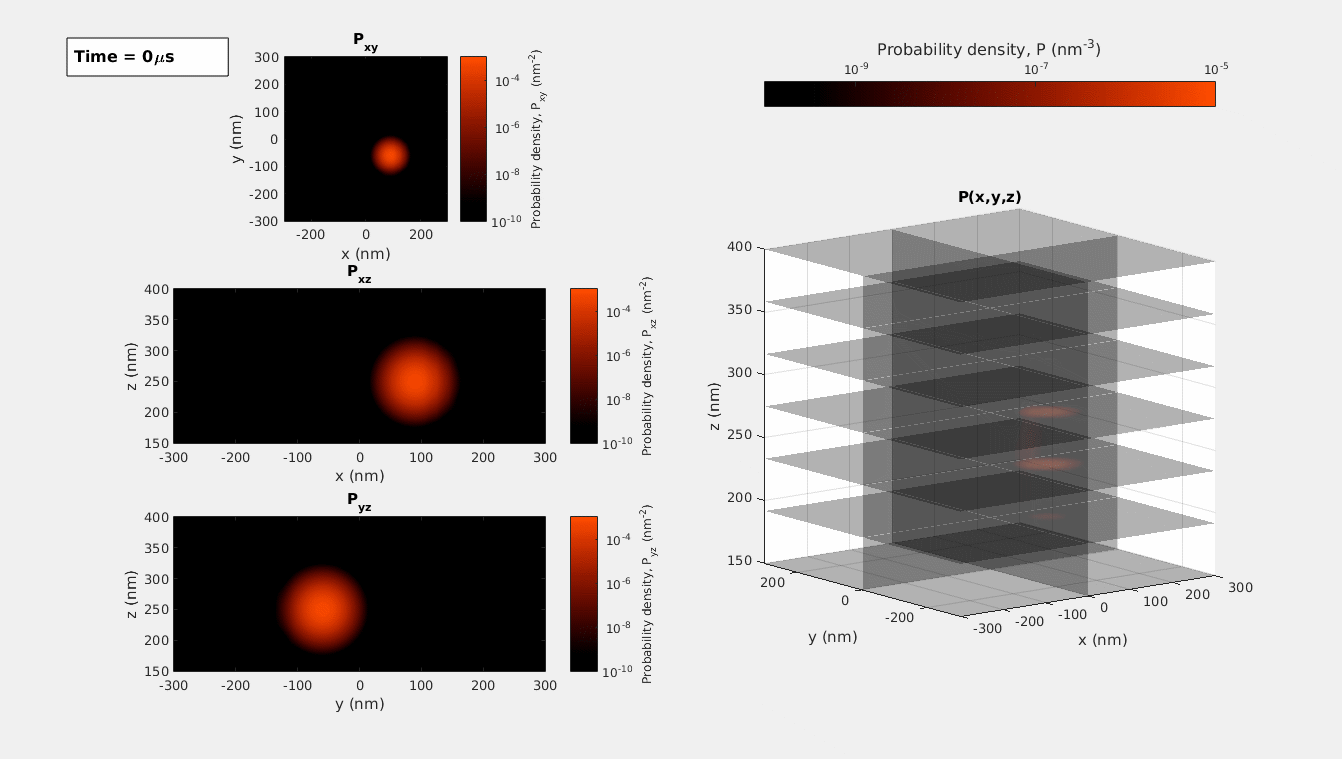

Supplement: Supplementary file 3 — Video file showing time evolution of PDF [file 41598_2019_45609_MOESM3_ESM.gif]

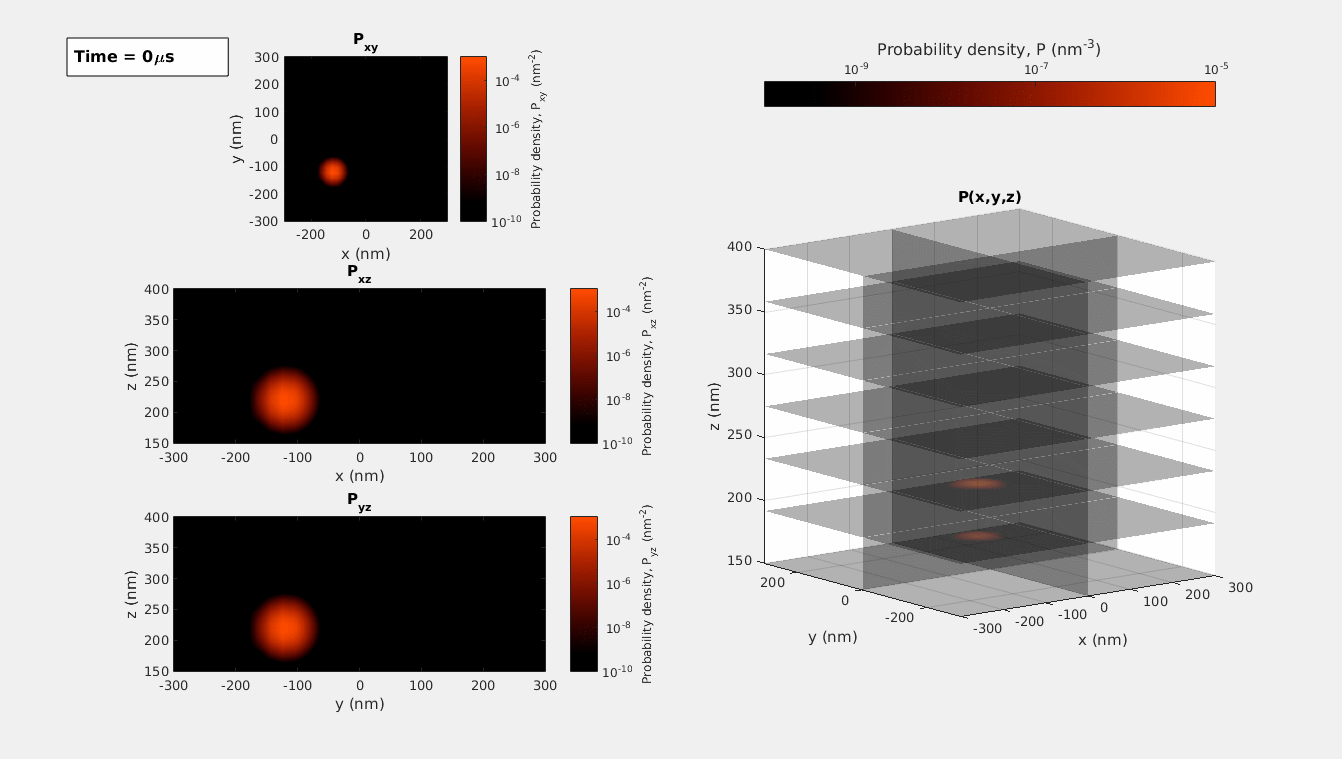

Supplement: Supplementary file 4 — Video file showing time evolution of PDF [file 41598_2019_45609_MOESM4_ESM.gif]

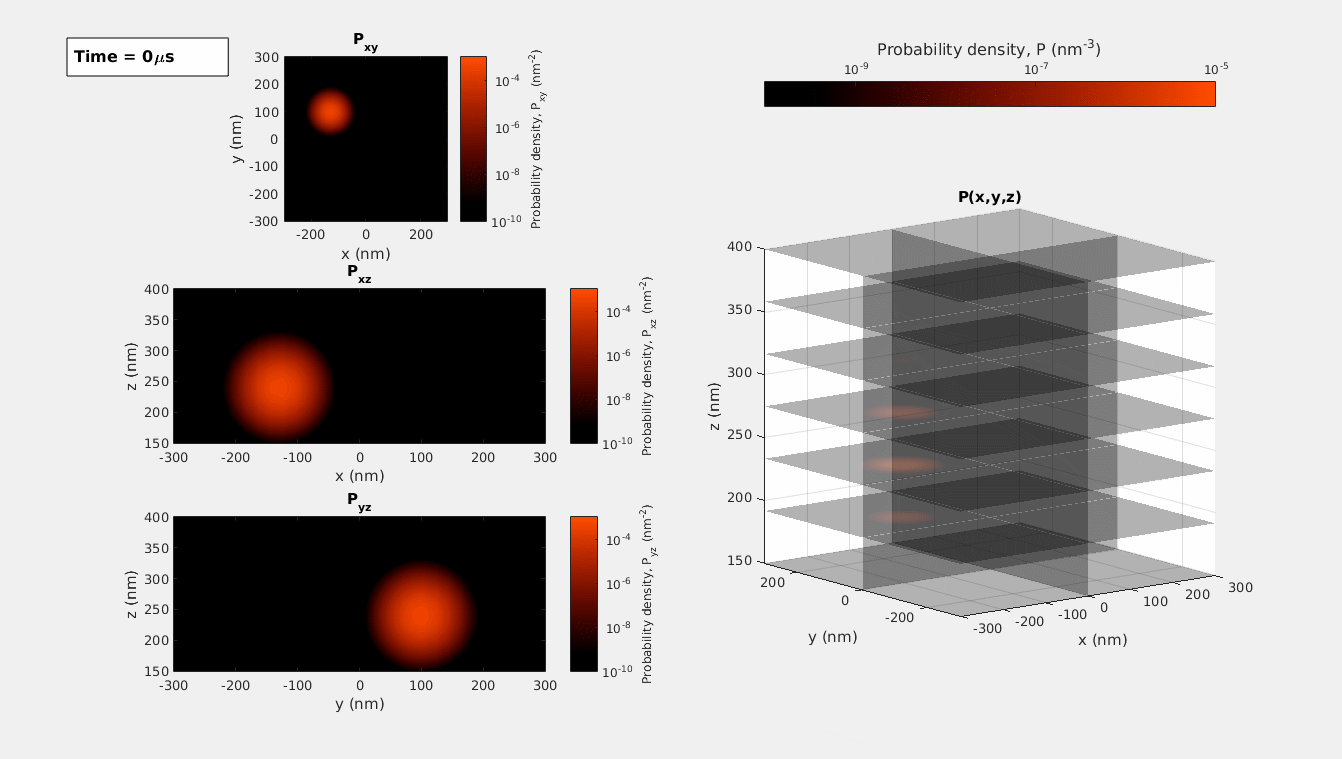

Supplement: Supplementary file 5 — Video file showing time evolution of PDF [file 41598_2019_45609_MOESM5_ESM.gif]
